# Toothache, tooth brushing frequency and dental check-ups in children and adolescents with and without disabilities

**DOI:** 10.25646/9565

**Published:** 2022-03-30

**Authors:** Laura Krause, Stefanie Seeling, Franziska Prütz, Julia Wager

**Affiliations:** 1 Robert Koch Institute, Berlin Department of Epidemiology and Health Monitoring; 2 German Paediatric Pain Centre, Children’s and Adolescents’ Hospital, Datteln; Department of Children’s Pain Therapy and Paediatric Palliative Care, Witten/Herdecke University, Faculty of Health, School of Medicine, Witten

**Keywords:** ORAL HEALTH, DENTAL HEALTH, UTILISATION, DISABILITIES, KIGGS

## Abstract

According to international studies, children and adolescents with disabilities have more tooth decay, brush their teeth less often twice a day and use preventive dental services less often than children and adolescents without disabilities. With data from the second follow-up to the German Health Interview and Examination Survey for Children and Adolescents (KiGGS Wave 2, 2014–2017), toothache, tooth brushing frequency and dental check-ups are examined in children and adolescents with and without disabilities. It was found that children and adolescents with disabilities had more toothache in the three months before the survey (23.5% and 15.9%, respectively) and brushed or got their teeth brushed twice a day less often (33.5% and 22.2%, respectively) than children and adolescents without disabilities. Differences in the utilisation of dental check-ups could not be determined. Overall, the results point to the importance of measures to promote tooth brushing frequency in order to improve the oral health of children and adolescents with disabilities. In addition, further opportunities should be created to collect data on the oral health of people with disabilities at the population level in health or participation studies.

## 1. Introduction

Oral health is a central component of general health and of great importance for well-being and quality of life [1]. Nationwide and population-representative data on oral health are available from the Fifth German Oral Health Study (DMS V), which was conducted by the Institute of German Dentists (IDZ) between 2013 and 2014 [2]. The data show that in 12-year-old adolescents, caries experience on permanent teeth has declined sharply in recent decades due to dental prophylaxis and good dental care. The data of the epidemiological accompanying studies on group prophylaxis, which are collected by the German Association for Youth Dental Care (DAJ), support this finding, but also show that caries experience in the primary dentition is still frequent among 3-year-olds in day-care centres and 6-to 7-year-olds in the first grade of school (13.7% and 43.6%, respectively) [3]. The highest caries prevalence is found in all age groups among children and adolescents from families in the low socioeconomic status group [2–4].

The presence of a disability can have a negative impact on oral health. For example, mental and psychological disabilities, but also some physical disabilities (e.g. hands or arms) and sensory impairments (e.g. visual impairments, hearing impairments) can be associated with poorer oral health [5]. Overall, there are very few studies on the oral health of children and adolescents with disabilities in Germany [5]. A few studies have examined the oral health of children and adolescents with disabilities in comparison to children and adolescents without disabilities [6–8]. The results indicate that, on average, children and adolescents with disabilities have a (significantly) higher caries experience than children and adolescents without disabilities [6–8]. Furthermore, isolated studies have examined the oral health of children and adolescents with disabilities without making a comparison to children and adolescents without disabilities [9–11]. According to the results, children and adolescents with intellectual and mental disabilities have significantly higher caries experience than children and adolescents with physical disabilities [9–11]. In this respect, the oral health of children and adolescents with disabilities varies depending on the type and severity of the disability [12].

**KiGGS Wave 2**

Second follow-up to the German Health Interview and Examination Survey for Children and Adolescents

**Data owner:** Robert Koch Institute

**Aim:** Providing reliable information on health status, health-related behaviour, living conditions, protective and risk factors, and health care among children, adolescents and young adults living in Germany, with the possibility of trend and longitudinal analyses

**Study design**: Combined cross-sectional and cohort study

**Cross-sectional study in KiGGS Wave 2**

**Age range:** 0–17 years

**Population:** Children and adolescents with permanent residence in Germany

**Sampling:** Samples from official residency registries - randomly selected children and adolescents from the 167 cities and municipalities covered by the KiGGS baseline study

**Sample size:** 15,023 participants

**KiGGS cohort study in KiGGS Wave 2**

**Age range:** 10–31 years

**Sampling:** Re-invitation of everyone who took part in the KiGGS baseline study and who was willing to participate in a follow-up

**Sample size:** 10,853 participants

**KiGGS survey waves**

► KiGGS baseline study (2003–2006), examination and interview survey
► KiGGS Wave 1 (2009–2012), interview survey
► KiGGS Wave 2 (2014–2017), examination and interview survey

More information is available at www.kiggs-studie.de/english

In order to prevent caries, in addition to a tooth-healthy diet and the use of fluorides (e.g. fluoride toothpaste for brushing teeth, fluoridated salt for meal preparation), appropriate dental care and regular dental check-ups are essential [13, 14]. In addition to frequency (at least twice a day), the duration of tooth brushing also plays a role in dental care; at least two minutes are recommended here, regardless of the type of toothbrush [14]. In the second follow-up to the German Health Interview and Examination Survey for Children and Adolescents (KiGGS Wave 2, 2014 –2017), data was collected on the frequency of tooth brushing and the utilisation of dental check-ups [15]. The analyses to date show that 22.3% of children and adolescents aged 0 to 17 years brush or get their teeth brushed less frequently than twice a day, and 19.7% present themselves for check-ups at a dental practice less frequently than twice a year. In families with low socioeconomic status, the figures are 40.3% (twice-daily tooth brushing) and 31.8% (twice-yearly dental check-ups) [15]. Whether children and adolescents with and without disabilities differ in their oral health behaviour has been rarely studied and only internationally [16, 17]. The studies provide evidence that children and adolescents with disabilities have less favourable oral health behaviour than children and adolescents without disabilities.

Against this background, this article describes the occurrence of toothache, the frequency of tooth brushing and the utilisation of dental check-ups in 3- to 17-year-old children and adolescents with and without officially recognised disability (in the following children and adolescents with and without disabilities, Info box 1) on the basis of data from KiGGS Wave 2 (2014–2017). Statements on the presence of caries cannot be made because no dental examination took place in KiGGS Wave 2. The indicator on toothache [18] is used as an indication of caries.

## 2. Methodology

### 2.1 Sample design and study conduct

KiGGS is part of the health monitoring system of the Robert Koch Institute (RKI) [23]. The KiGGS baseline study (2003–2006) provided population-based, nationally representative results on the health situation of 0- to 17-year-old children and adolescents in Germany for the first time. With KiGGS Wave 2 (2014–2017), a good ten years later, the most up-to-date data is available. Those to be invited were randomly drawn from the population registers in 167 cities and communities that were representative of Germany and had already been selected for the baseline study. A variety of measures were used to achieve a high number of participants as well as a sample that corresponds as closely as possible to the composition of the population [24]. A total of 15,023 children and adolescents participated in KiGGS Wave 2 (response: 40.1%). The concept and design of the study are described in detail elsewhere (see also Info box on KiGGS Wave 2) [24, 25].

**Info box 1 Children and adolescents with disabilities**

‘People with disabilities are hindered in activities of daily living and/or equal participation by interactions of their own impairments and accessibility in the environment’ [19]. People with a recognised disability are ‘all persons whose disability has been determined or recognised by a competent office’ [19]. This includes the awarding of a severity grade of disability (GdB). The GdB can be between 20 and 100, whereby a GdB ≥50 is classified as severe disability.

According to the microcensus, 216,000 children and adolescents under 18 years of age with an officially recognised disability lived in private households in 2019 [20]. This corresponds to a share of 1.6% of all under 18-year-olds in Germany. According to the statistics on severe disabilities, 194,213 children and adolescents under the age of 18 had an officially recognised severe disability [21]. About three quarters of these were caused by illness (77.3%), almost one fifth were congenitally disabled (18.7%) and 4% had other causes (including accidents) [21]. The most common forms of severe disability among under 18-year-olds in 2019 were mental disabilities/learning disabilities (68,041), followed by physical disabilities (54,864). Speech and language disorders, deafness, hearing loss and balance disorders ranked third (8,569), mental disabilities fourth (6,843) and blindness and visual impairments fifth (5,041) ([22], own calculations).

### 2.2 Description of the indicators

Information on the indicators used here was collected in a questionnaire to be completed in writing. The question on the existence of an officially recognised disability was only asked of the parents or guardians. The questions on toothache, tooth brushing frequency and utilisation of dental check-ups were answered by the guardians for up to 10-year-olds, while 11- to 17-year-olds provided information themselves. For 11- to 17-year-olds with an officially recognised disability, the answers were given by the guardians. Since the question on dental pain was not asked of the guardians of children between 0 and 2 years, the data basis in this article refers to the age group of 3 years and older.

The question on the existence of an officially recognised disability was: ‘Does your child have a disability officially recognised by the pension office? The possible answers were ‘yes’ and ‘no’. Regarding pain, the question was: ‘Has your child/have you had the following pain in the last 3 months?’ ‘Tooth’ could be ticked in a list of given locations [26]. The three-point response scale was divided into ‘yes, once/yes, repeatedly’ and ‘no’ for the analyses. The question on tooth brushing frequency was: ‘How often are your child’s teeth brushed or how often does your child brush his/her teeth?’ or ‘How often do you brush your teeth?’ Response options were ‘twice a day or more often’, ‘once a day’, ‘several times a week’, ‘once a week or less often’ and ‘not at all’ (multiple responses were not possible). For the analyses, the answer option ‘twice a day or more often’ was compared to the other categories [15]. Regarding the use of dental check-ups, the question was: ‘At what intervals does your child go to the dentist for a check-up?’ or ‘At what intervals do you go to the dentist for a check-up?’ Response options were ‘quarterly’, ‘every six months’, ‘once a year’, ‘less often’ and ‘I have never been to the dentist’. For the analyses, the response categories were combined into ‘quarterly/semi-annually’ versus the other options [15].

### 2.3 Statistical analysis

The analyses are based on data from 13,308 children and adolescents aged 3 to 17 years with a valid response to the question about an officially recognised disability (yes/no). Table 1 shows the distribution of the sample based on important sociodemographic characteristics. Since in KiGGS Wave 2 a disability was only indicated for 261 children and adolescents (n=47 participants with a degree of disability (GdB) <50; n=177 with a GdB ≥50; n=37 missing information on GdB), no stratifications by degree and form of disability or other characteristics such as sex and age are possible.

**Info box 2 Socioeconomic status of the family**

In KiGGS,, the socioeconomic status of the family is determined on the basis of information provided by the parents on their school education and professional qualifications, their professional position and the needs-weighted net household income. Based on an index formed as a sum of point scores, in which the three indicators are included with equal weighting, a distribution-based delimitation of three groups is carried out, according to which 20% of children and adolescents are to be assigned to the low (first quintile), 60% to the middle (second – fourth quintile) and 20% to the high socioeconomic status group (fifth quintile) [29].

Prevalence and univariate and multivariate prevalence ratios (PR) with 95% confidence intervals were calculated from log-Poisson regressions, with the presence of a disability as the dependent outcome variable. The multivariate regression analyses for toothache, tooth brushing frequency and utilisation of dental check-ups were adjusted for sex, age and family socioeconomic status (Info box 2). Due to the strong association between oral health and the presence of a disability [5] on the one hand and the social situation [27] on the other hand, an interaction between disability and socioeconomic status was taken into account in the multivariate regression analyses in a further step. Furthermore, in the multivariate regression model for toothache, the frequency of tooth brushing and the utilisation of dental check-ups were additionally controlled for. A statistically significant difference between children and adolescents with and without disabilities is assumed if the p-value is smaller than 0.05.

All analyses were conducted using the survey procedures of Stata 17.0 (Stata Corp., College Station, TX, USA, 2015) in order to take the cluster design of KiGGS and the weighting appropriately into account when calculating confidence intervals and p-values. The analyses were calculated with a weighting factor that corrects for deviations of the sample from the population structure with regard to regional structure (city/rural), age (in years), sex, federal state (as of 31.12.2015), German citizenship (as of 31.12.2014) and parental education (Microcensus 2013 [28]).

## 3. Results

**Figure 1:**
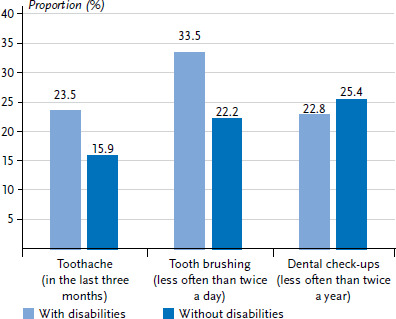
Toothache (once/repeated), tooth brushing frequency and utilisation of dental check-ups in 3- to 17-year-olds (n=261 children and adolescents with disabilities, n=13,047 children and adolescents without disabilities) Source: KiGGS Wave 2 (2014–2017)

Figure 1 shows that 3- to 17-year-old children and adolescents with disabilities were affected by single or recurrent toothache more frequently in the three months prior to the survey than their peers without disabilities (23.5% and 15.9%, respectively). They were also less likely to brush their teeth twice a day (by themselves or a caregiver) (33.5% and 22.2%, respectively). There were no differences between children and adolescents with and without disabilities in the utilisation of dental check-ups (22.8% and 25.4%, respectively). The differences between children and adolescents with and without disabilities in the occurrence of toothache and in tooth brushing frequency are statistically significant, as shown by univariate log-Poisson regression models (toothache: univariate PR 1.5, p=0.023; tooth brushing frequency: univariate PR 1.5, p=0.005; data not shown).

The association between tooth brushing frequency and the presence of a disability remained after controlling for age, sex and socioeconomic status of the family in the multivariate log-Poisson model (multivariate PR 1.3, p=0.020). If an interaction between disability and socioeconomic status was additionally considered in this model, it was shown that it was especially children and adolescents with disabilities from families in the low socioeconomic status group for whom twice-daily toothbrushing occurred less frequently (multivariate PR 4.3; p=0.028; data not shown).

The association between toothache and disability was no longer significant after statistically controlling for all characteristics (multivariate PR 1.4; p=0.070; data not shown). If, in addition to age, sex and socioeconomic status, tooth brushing frequency and the utilisation of dental check-ups were included in the multivariate model for toothache, it was shown that in particular too little tooth brushing frequency (less than twice a day) and a low socioeconomic status of the family explained the occurrence of toothache in children and adolescents (multivariate PR <0.001 each; Table 2).

## 4. Discussion

The aim of this study was to identify possible differences in the occurrence of toothache, in the frequency of tooth brushing and in the utilisation of dental check-ups between children and adolescents with and without disabilities based on data from a representative sample for Germany.

Children and adolescents with disabilities were affected by single or recurrent toothache more frequently in the last three months than children and adolescents without disabilities. This result is in line with international studies [30, 31]. A very common cause of toothache is caries [18]. Thus, the higher caries experience among children and adolescents with disabilities described in the literature also fits our finding [6, 7]. However, toothache cannot only be equated with a manifest (i.e. untreated) caries, as it can also occur, for example, with teeth that have already been treated, with eruption disorders in the wisdom tooth region and, under certain circumstances, during the change of teeth. In addition, caries that has been present for a long time can also be associated with reduced pain sensitivity. In the multivariate model on toothache, it was shown that there is no direct correlation between toothache and disability in childhood and adolescence, but that this is primarily mediated by an insufficient frequency of tooth brushing (less frequently than twice a day) and a low socioeconomic status of the family. In this context, studies should be considered that provide evidence that persons with a low socioeconomic status compared to persons with a high socioeconomic status may have a higher perception of pain and a lower individual pain threshold [32], which may be related to a lack of coping strategies and a low self-efficacy experience [33]. This could possibly also play a role in people with certain disabilities.

A direct correlation was shown between tooth brushing frequency and the presence of a disability: twice-daily tooth brushing occurred less frequently among children and adolescents with disabilities; this is particularly true for children and adolescents with disabilities from families in the low socioeconomic status group. To the authors’ knowledge, there are only a few studies that have investigated the tooth brushing frequency of children and adolescents with disabilities compared to children and adolescents without disabilities. A Dutch study [16] found that 16- to 18-year-olds with mild mental retardation and learning disabilities in special schools hardly differed from 17-year-olds in the general population in terms of tooth brushing frequency (75% and 76%, respectively). The reason discussed was whether social desirability might have played a greater role in the response behaviour of adolescents with mild mental retardation and learning disabilities than for adolescents from the general population [16] (for social desirability bias in tooth brushing frequency, see e.g. [34]). Despite brushing their teeth with equal frequency, the adolescents with mild intellectual disability and learning disability had poorer oral health status, which may indicate lower motor skills to perform tooth brushing (i.e. although teeth were brushed twice a day, bacterial plaque was not adequately removed) [16]; in particular, deficits in hand dexterity in children and adolescents with mild intellectual disability and learning disability are described in the literature [35]. A Saudi Arabian study [17] points in a similar direction as the KiGGS study – taking into account sociocultural differences in oral health behaviour [36] – according to which 6- to 12-year-old girls with visual impairments at special schools were less likely to report brushing their teeth daily than girls without visual impairments at primary schools (78.5% and 90.4%, respectively). Furthermore, isolated studies have examined the oral health of children and adolescents with disabilities without making a comparison to children and adolescents without disabilities [37–41]. These studies consistently show that the majority of children and adolescents with disabilities brush or get their teeth brushed less frequently than twice a day. Reasons for the lower frequency of tooth brushing among children and adolescents with disabilities may include limited communication and cooperation skills in oral hygiene, in addition to the already mentioned lower mental and/or motor skills to perform tooth brushing [42]. Pathological biting reflexes and head movements, which affect some children and adolescents with specific disabilities, can also make brushing teeth difficult. Further barriers on the part of the parents may be low oral health awareness or a high physical and psychological burden of daily care [42].

About a quarter of children and adolescents with and without disabilities have attended less than two dental check-ups within one year. This means that about three quarters (77.2% of children and adolescents with and 74.6% of those without disabilities) have presented themselves to the dental practice for a check-up at least twice a year. In Germany, children and adolescents with an increased caries risk can even make utilisation of dental check-ups and preventive measures four times a year on the basis of the statutory health insurance (SHI). Looking at the proportion of children and adolescents who attended quarterly dental check-ups, there is a weak but statistically significant difference in favour of children and adolescents with disabilities (18.4% and 12.0%, respectively; univariate PR 1.5, p=0.040; multivariate PR 1.5, p=0.049 (adjusted for age, sex and socioeconomic status); data from this sensitivity analysis are not shown). These findings are in contrast to the results of international studies: in the previously cited work from the Netherlands [16], the proportion of adolescents consulting a dentist twice a year among those with mild mental retardation and learning disabilities in special schools was only about half as high at 44% as among adolescents from the general population at 82%. In the study from Saudi Arabia [17], the proportion of 6- to 12-year-old girls who regularly visited a dental practice was more than 15 percentage points lower among those with visual impairments in special schools than among girls without visual impairments in primary schools (54.5% and 71.0%, respectively). Studies that have investigated dental utilisation exclusively among children and adolescents with disabilities at special schools uniformly point to a very low utilisation of preventive dental services by children and adolescents with disabilities [38–40]. However, international comparisons of the utilisation of health services are only of very limited value due to the different health and social systems. Reasons for lower dental utilisation by children and adolescents with disabilities may be, for example, a lack of wheelchair access to the dental practice or difficulties in finding a dental practice where children and adolescents with disabilities can be adequately cared for [42].

When surveying utilisation in population-based studies such as the RKI health surveys, it should be noted that certain groups of people, such as very ill or severely impaired people, are underrepresented in surveys [43]. It can be assumed that something similar also applies to parents of children with disabilities. Therefore, the present results should be interpreted with caution. There may be differences in the utilisation of dental check-ups between children and adolescents with and without disabilities that could not be identified in this study. This assumption is supported by the results of a study which, based on billing data from the National Association of Statutory Health Insurance Dentists ((Kassenzahnärztliche Bundesvereinigung, KZBV), was able to show that individual prophylactic measures are carried out and billed less frequently for children and adolescents who have a care degree or receive integration assistance than for those without a care degree and without receiving integration assistance [44]. Differences in the occurrence of toothache and in tooth brushing frequency may also be underestimated due to the selectivity of the sample (see also [20]). Further limitations may be recall bias [45] or responses in terms of social desirability [34], which may be different for children and adolescents with and without disabilities. Furthermore, it should be noted that children and adolescents with disabilities are a heterogeneous group with very different health situations and needs, which could not be surveyed in detail in the KiGGS study.

## Conclusion and outlook

According to the results of KiGGS Wave 2, children and adolescents with disabilities had toothache more often than children and adolescents without disabilities. The relation between toothache and disability is mainly explained by a low socioeconomic status and a too low tooth brushing frequency: Children and adolescents with disabilities brushed or got their teeth brushed less frequently than twice a day; this applies in particular to children and adolescents with disabilities from families in the low socioeconomic status group. To prevent caries, it is important that children and their parents are made aware at an early age to brush their teeth at least twice a day [46]. Special attention should be paid not only to families of the low socioeconomic status group [46], but also to children and adolescents with disabilities. Since children and adolescents with disabilities also generally brush their teeth less efficiently [47], measures to improve tooth brushing behaviour are of great importance. An international study was able to show that children and adolescents do not benefit equally from measures to improve tooth brushing behaviour, depending on the type of disability: Children and adolescents with intellectual disabilities did not show any improvement in tooth brushing behaviour in the context of an intervention, whereas children and adolescents with physical disabilities and sensory impairments did [48]. In this respect, interventions for more effective tooth brushing should be developed according to the type and severity of the disability [49]. Dentists play an important role in teaching tooth brushing, as they offer advice and instructions on oral hygiene as preventive services and the costs for these are also covered by the SHI in Germany. It is important to also address and improve the motor skills of children and adolescents with disabilities [35]. Close cooperation with parents and caregivers is essential, as the oral health of children and adolescents with disabilities is decisively influenced by their knowledge of effective oral hygiene [50].

According to the results from KiGGS Wave 2, children and adolescents with and without disabilities make utilisation of dental check-ups with equal frequency: Only about a quarter of them visited a dental practice for a check-up less frequently than twice a year. In view of the fact that children and adolescents with disabilities have an increased caries risk, it is necessary to consider how the dental care of this very heterogeneous patient group can be further improved. In addition to the accessibility of dental practices, the topic of care for people with disabilities must also be addressed in postgraduate dental training in order to ensure demand-oriented care [5, 42]. If access to dental care is limited for people with disabilities, outreach public dental health services can provide support [50]. Therefore, in addition to dental care, group prophylactic care in schools and inclusive institutions by community dental services is of great importance for the dental health equity of children and adolescents with disabilities.

The present results point to a need for further research on oral health and oral health behaviour of children and adolescents with disabilities in Germany, also against the background of the incomplete data situation. It would be conceivable to survey dental findings within the framework of health or participation studies, to survey disabilities in oral health studies or to design a separate study on the oral health of people with disabilities. In general, the presence of a disability should be taken into account in health studies, in addition to the usually used stratification characteristics such as age, sex and socioeconomic status of the family, if possible due to the number of cases [51].

## Key statements

- Children and adolescents with disabilities were affected by single or recurrent toothache more frequently than children and adolescents without disabilities.
- Compared to children and adolescents without disabilities, children and adolescents with disabilities had a lower daily frequency of tooth brushing.
- There were no differences between children and adolescents with and without disabilities in the utilisation of dental check-ups.

## Figures and Tables

**Table 1:** Sample description (n=261 children and adolescents with disabilities, n=13,047 children and adolescents without disabilities) Source: KiGGS Wave 2 (2014–2017)

Number of cases (n)		Unweighted sample (%)	Weighted sample (%)
**Children and adolescents with disabilities**			
**Sex**			
Girls	125	47.9	46.9
Boys	136	52.1	53.1
Age group^*^			
3–10 years	121	46.4	49.9
11–17 years	140	53.6	50.1
**Socioeconomic status**			
Low	54	21.3	30.9
Medium	150	59.1	57.5
High	50	19.7	11.6
**Children and adolescents without disabilities**			
**Sex**			
Girls	6,555	50.2	48.5
Boys	6,492	49.8	51.5
Age group^*^			
3–10 years	6,753	51.8	51.5
11–17 years	6,294	48.2	48.5
**Socioeconomic status**			
Low	1,595	12.3	19.8
Medium	8,030	61.9	60.6
High	3,354	25.8	19.6

^*^ The mean age for children and adolescents with disabilities is 10.4 years (95% CI 9.7–11.2), for children and adolescents without disabilities 10.2 years (95% CI 10.1–10.3)

**Table 2:** Toothache in the last three months (once/repeated) according to sociodemographic and dental factors in 3- to 17-year-olds (n=261 children and adolescents with disabilities, n=13,047 children and adolescents without disabilities) Source: KiGGS Wave 2 (2014–2017)

Prevalence Ratio* (95% CI)		p-value
**Disability**		
No	Ref.	–
Yes	1.4 (0.9–2.0)	0.102
**Age group**		
3–10 years	Ref.	–
11–17 years	1.1 (1.0–1.3)	0.036
**Sex**		
Girls	Ref.	–
Boys	0.9 (0.8–1.0)	0.016
**Socioeconomic status**		
Low	1.5 (1.3–1.8)	<0.001
Medium	1.0 (0.9–1.2)	0.526
High	Ref.	–
**Tooth brushing (twice daily)**		
Yes	Ref.	–
No	1.3 (1.2–1.6)	<0.001
**Dental check-ups (twice yearly)**		
Yes	Ref.	–
No	0.9 (0.7–1.0)	0.031

CI = Confidence interval, Ref. = Reference group

^*^ Results from multivariate log-Poisson regressions
